# Effects of different mulch materials on the photosynthetic characteristics, yield, and soil water use efficiency of wheat in Loess tableland

**DOI:** 10.1038/s41598-023-45309-7

**Published:** 2023-10-23

**Authors:** Shenglan Ye, Juan Li, Hui Kong, Jianglong Shen, Dan Wu

**Affiliations:** 1 Institute of Land Engineering and Technology, Shaanxi Provincial Land Engineering Construction Group Co., Ltd., Xi’an, 710075 China; 2 Shaanxi Provincial Land Engineering Construction Group Co., Ltd., Xi’an, 710075 China; 3 Key Laboratory of Degraded and Unused Land Consolidation Engineering, Ministry of Natural Resources, Xi’an, 710075 China

**Keywords:** Biogeochemistry, Plant physiology

## Abstract

Due to the lack of precipitation and poor temporal and spatial stability in the Loess Plateau for a long time, it is necessary to adopt mulching technology to support the stable and high yield of wheat cultivation system. This study aims at exploring different mulching materials on the soil water content, photosynthetic characteristics, wheat yield, and yield components of winter wheat in the gully region of the Loess Plateau. The results showed that the traditional flat soil water content is the lowest in the seedling stage. In the jointing stage and heading stage of many crop water requirements, ridge film mulching treatment can effectively promote the growth of wheat and increase the water use efficiency. The leaf area index (LAI) of different treatments wheat showed a trend of increasing first and then decreasing. In the jointing stage, ordinary mulching film (T1) and liquid mulching film (T3) had the highest LAI content, which were 3.78 and 3.71 respectively. The Pn and Gs in wheat flag leaves of T3 treatment is higher than that of CK throughout the entire growth period, and T3 significantly increased Ci and *WUE*i in different growth stage. And the grain number per panicle and grain weight of T3 treatment were 24.44 and 41.00 g, which were 19.3% and 5.4% higher than CK, respectively. Through the actual production calculation of the final harvest, the ridge film mulching has a significant increase in production compared with the CK. The yield of T3 treatment was 4980.25 kg hm^−2^, which was 29.37% higher than CK. It was significantly different from CK (*P* < 0.05). Based on the comprehensive analysis, the ridge film mulching treatment significantly affected the soil water content and wheat yield. And the liquid mulching film had the best effect. Exploring the impact of different covering techniques on the wheat cultivation system in the Loess Plateau region, to promote the scientific promotion of this technology.

## Introduction

Water deficit is the main factor restricting the development of dryland agriculture^1^. Dryland agriculture accounts for about 55% of China's total arable land, which is mainly concentrated in northern China^2^. The northern dryland area is also the main potential area for grain production in China in the future. Wheat (*Triticum aestivum* L.) is the most widely planted crop in northern China. There are 4.3 million to 4.7 million hectares of planting area in dryland areas, and the annual precipitation in dryland wheat production areas in northern China is between 250 and 600 mm^3^. In this region, 55% to 60% of the precipitation occurs from June to September. The winter wheat growth season has scarce precipitation, strong inland climate, large soil evaporation, and frequent seasonal droughts^4^. Among them, Fuping County is in the northern part of the Weibei dry plateau, which belongs to the gully region of the Loess Plateau in northern China. The average annual rainfall is 533.2 mm^5^. Mulching is the main cultivation method for drought resistance, water saving, efficient water use, yield increase and harvest protection in rain-fed agricultural areas and non-protected irrigation areas^6,7^.

Plastic film mulching and straw mulching are the two most important mulching cultivation methods in China. Among them, ridge mulching technology is the most used mulching technology in the semi-arid region of northwest China^8,9^. Ridge film mulching technology has been widely used in agricultural production in semi-arid areas of my country. It can effectively retain water and increase crop yields. This technology has been widely used in different crops in Northwest China^10^. The covering material forms an atmosphere-soil heat exchange isolation layer in the topsoil, which reduces the daily range and annual range of soil temperature, thus creating relatively stable hydrothermal conditions for crop root growth. Qin et al.^11^ conducted a meta-analysis of 118 published studies on wheat mulching on the Loess Plateau since 2000. The results showed that the average yield increase rate of plastic film mulching was 4.04% higher than that of straw mulching. Chen et al.^12^ studied winter wheat in the dry farming area of the Loess Plateau in Northwest China and found that the yield increase efficiency of full film mulching was higher than that of full ground straw mulching and increased by 13% and 9% respectively compared with no mulching. Qin et al.^13^, a meta-analysis of 165 literatures on wheat mulching cultivation in northern China in the past 40 years showed that straw mulching and plastic film mulching significantly increased wheat yield by 19.5% and 24.9%, respectively. Plastic film mulching has more advantages in increasing yield in cold and arid regions. Therefore, this study chose plastic film mulching.

China is the largest country in the application of plastic film mulching, accounting for about 60% of the world 's agricultural film consumption^14^. Since ordinary plastic film and black plastic film are mainly polyethylene, it is difficult to fully recover the residual film after use. The pollution caused by soil and environment is becoming more and more serious, and it has many adverse effects on crop production. General plastic film mulching has begun to cause widespread criticism^15,16^. Due to the resistance to degradation of ordinary plastic film, when the accumulation in the soil exceeds a certain range, there will be many effects, including reducing soil permeability, porosity, permeability and absorption capacity of water and nutrients^17^; water use efficiency (WUE) and crop yield also decreased with the accumulation of residual film^14^. Therefore, it is urgent to find a new type of plastic film to replace ordinary plastic film coverage. Degradable film can effectively solve the problem of farmland pollution and provide technical support and theoretical basis for the sustainable development of agriculture in arid areas on the premise of ensuring water use efficiency and yield^18–20^. Wang et al.^21^ studied the application of flax planting in Qinghe County, Inner Mongolia at an altitude of 1300–1700 m. The results showed that spraying liquid film could effectively improve the yield and income of flax, and the productivity per plant increased by 0.34 g. Zhang et al.^22^ showed that the soil temperature of 5 ~ 15 cm soil layer after spraying liquid film was 4 °C higher than that of the control, the emergence rate of maize was 17% higher than that of the control, and the yield was 17.4% higher than that of the control. Different coverage technologies will have diverse impacts on wheat physiology, farmland microclimate, and even sustainable agricultural development. Adopting a suitable covering cultivation mode can improve soil quality, optimize the water consumption structure of wheat, and promote high and stable yield of wheat. Studying the effects of wheat mulching cultivation in this region can provide a scientific basis for the precise promotion of mulching technology. Therefore, this study compared and analyzed the effects of degradable biofilm and common plastic film on soil moisture content, yield components and wheat yield by using degradable biofilm and common plastic film as mulching materials. Finally, the advantages of degradable biofilm in agricultural production were clarified. At the same time, biofilm can also reduce the cost of recycling and reduce environmental pollution, which is of great significance to the ecological environment and economic benefits. This study provides an effective theoretical basis for the establishment of a reasonable mulching planting pattern in dryland areas.

## Materials and methods

### Overview of the study area

**Figure 1:**
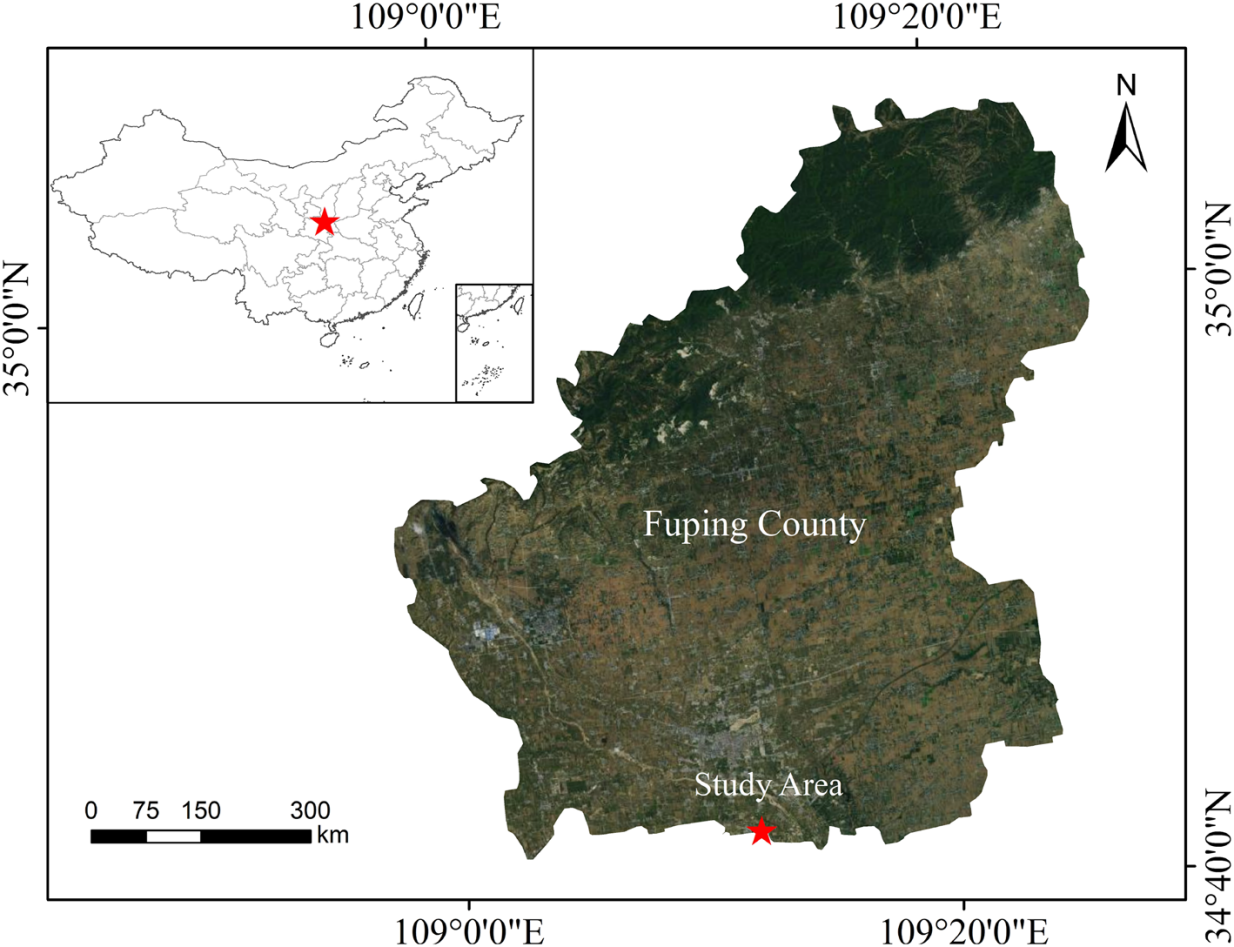
Geographical location of test area.

**Figure 2:**
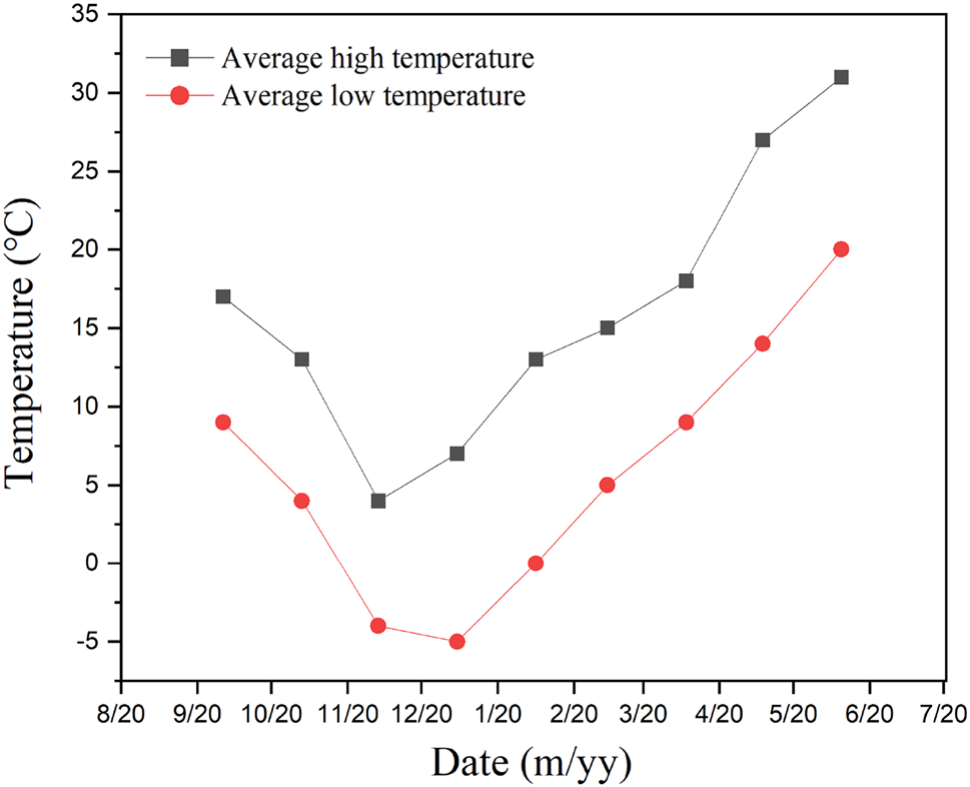
Temperature changes during wheat planting period (2020–2021).

The field test was carried out continuously in Fuping Middle trial site of the Institute of Land Construction from 2017 to 2021 (Fig. 1). The experimental data was collected after planting winter wheat in October 2020. The geographical location of the test area is shown in Fig. 1. The experimental land is provided by Shaanxi Land Engineering Construction Group. It is located at 34°42′ ~ 35°06′ north latitude and 108°57′ ~ 109°26′ east longitude. It belongs to the Loess tableland. The area has an altitude of 376–439 m. An average annual rainfall was 533.3 mm; An average annual temperature was 13.1 °C; An average annual sunshine time was 2472 h. And a frost-free period was 225 days. It belongs to a warm-temperate continental climate and crops are ripe for one year. The soil in the test area was loessal soil. The basic physical and chemical properties of soil are shown in Table 1 and Table 2. The chemical fertilizers were applied with pure N 150 kg hm^−2^, P_2_O_5_ 120 kg hm^−2^, and K_2_O 90 kg hm^−2^ before planting. The effective rainfall during the wheat growth period (October 2020 to June 2021) in the experimental year is shown in Table 3, and the temperature changes are shown in Fig. 2.

**Table 1:** Soil particle composition.

Layers (cm)	Clay (%)	Silt (%)	Sand (%)	Texture (USDA)
	< 0.002 mm	0.02–0.002 mm	2–0.02 mm	
0–30	1.3	73.3	25.4	Silty loam soil

**Table 2:** Soil nutrient content.

Layers (cm)	pH	Available P (mg kg^−1^)	Available K (mg kg^−1^)	Organic matter (g kg^−1^)	Total N (g kg^−1^)
0–30	8.34	7.9	91.4	8.81	0.68

**Table 3:** Rainfall during the growth period of winter wheat from 2020 to 2021.

Month	Oct	Nov	Dec	Jan	Feb	Mar	Apr	May	Jul	Total
Total rainfall	63.2	21.4	1.3	27.2	10.5	12.5	27.5	30.8	10.6	205.0
Effective rainfall	57.8	15.7	0	27.2	9.3	12.5	27.5	28.9	10.6	189.5

### Test materials and design

**Figure 3:**
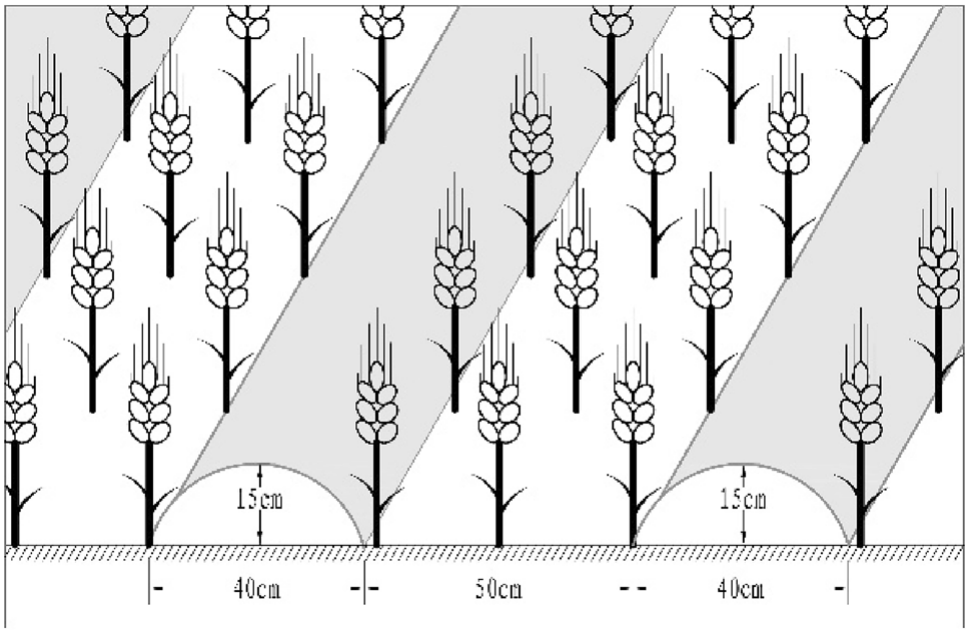
Ridging film mulching planting mode.

**Figure 4:**
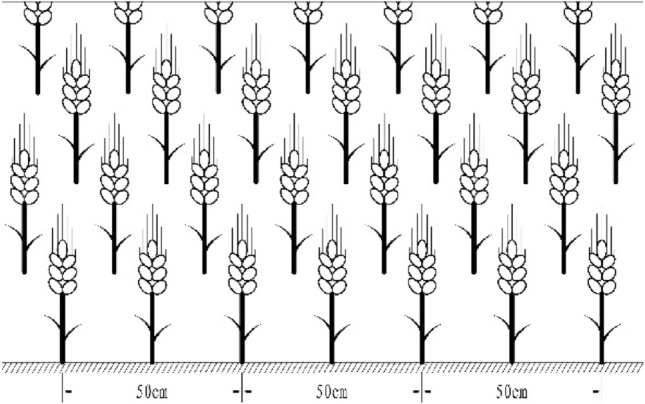
Traditional flat planting mode.

The tested material is winter wheat (Triticumaes-tivum) Xiaoyan 22 which was purchased from Shaanxi Fuping County Seed Industry Co., Ltd. On October 9, 2020, the seeds will be sown according to the local production rate, and the drill will be sowed. The seeding rate is 150 kg hm^−2^, and the row spacing is 25 cm. All harvested on June 2, 2021. Flood irrigation is carried out during the period when crops are in large water demand. The irrigation volume is 48 mm. In this experiment, the mulching width is 40 cm, the ridge height is 15 cm, and the furrow is 50 cm. The planting area is not covered with plastic film (Fig. 3). The control (CK) is a traditional flat crop, without film mulching and no ridges (Fig. 4); T1 treatment is ordinary mulching film; T2 treatment is black mulching film; T3 treatment is liquid mulching film that can be degraded biofilm. The experiment consists of 4 treatments, with each treatment repeated 4 times. The area of the test plot is 3 × 210 + 200 = 830 m^2^. Ordinary plastic film is made of polyethylene as the main raw material, with the addition of antioxidants, which can retain water, fertilizer, and insulation. Black plastic film, with the addition of black masterbatch in the production process of ordinary plastic film, has stronger insulation ability. Biodegradable liquid plastic film is a polymer material formed by special processing of natural polymer substances such as lignin, collagen, surfactants, and soil water retention agents, using crop straw as raw materials. It is a new type of environmentally friendly plastic film.

### Measurement items and methods

*Soil moisture content* In the critical period of crops, the soil drilling method is used to measure the soil moisture content of each treatment 0–100 cm soil layer, the sampling interval is 10 cm, and the soil is drilled and dried and weighed to calculate the soil moisture content.

*Leaf area index* In the critical period of crops, the LAI2200 leaf area index instrument is used to determine the wheat leaf area index.

*Photosynthetic parameter determination*^23^ On a sunny day from 9:00 to 11:30, the Li-6400XT portable photosynthesis measurement system (Li Cor, USA) was used to configure leaf chambers 6400-11 to measure the photosynthetic rate (Pn), stomatal conductance (Gs), intercellular CO_2_ concentration (Ci), and transpiration rate (Tr) of wheat flag leaves. Each treatment was repeated 3 times, and the instantaneous water use efficiency (WUEi) was calculated. WUEi = Pn/Tr.

*Water use efficiency* *WUE* = *Y*/*ET*, where *WUE* is the water use efficiency of yield, kg‧(hm^−2^ mm^−1^), Y is the wheat yield, kg hm^−2^, and ET is the average water consumption of wheat (mm). The required meteorological data is obtained from Fuping County Weather Station.

*Growth period time* Through observation and record the specific time of different key growth periods of wheat.

*Yield and yield components* After the wheat matures, each plot selects three samples of 2 m^2^ for indoor roasting, and measures the wheat spike number, spike grain number, and 1000-grain weight. The output is calculated based on the actual harvest in the test area.

Biomass: 0.25 m^2^ wheat plants are selected in each critical period, and their plant height, wet weight and dry weight are determined to determine their dry weight.

### Statistical analysis

The experimental data were sorted and calculated by Microsoft Excel 2010, and the significant difference test was carried out by LSD method. The significant level was set to α = 0.05, and the correlation between biomass and LAI was analyzed by Pearson correlation analysis. The data were analyzed by SPSS 16.0. ArcGIS 10.7 and origin2021 was used for drawing.

## Results and analysis

### Effect of different film mulching planting on soil water content

**Figure 5:**
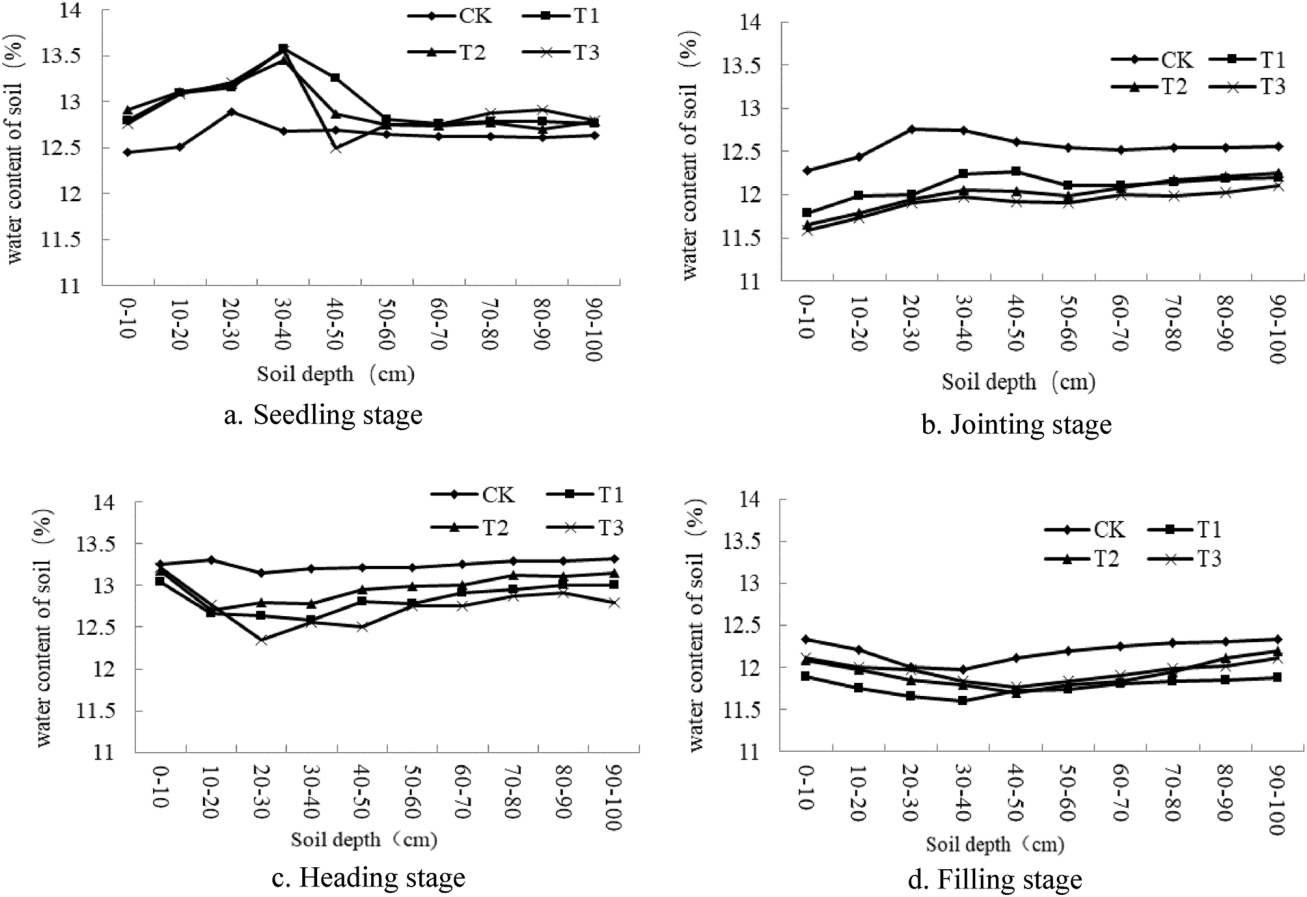
Soil water content of different growth stages.

It can be seen from Fig. 5 that there are significant differences in the soil water content of different treatments at different growth periods of wheat. In Fig. 1a, the soil moisture content reached the highest at 30–40 cm in each treatment. T1, T2 and T3 were 13.56%, 13.44% and 13.58%, respectively. And the difference between the three is not significant. The soil moisture content of traditional flat cropping reaches the highest at 20–30 cm. And CK was significantly lower than other treatments, only 12.89%. The traditional flat cropping soil water content in Fig. 1b–d is higher than other ridge mulching treatments. The main reason is that the rapid growth of crops during the critical period of wheat growth requires a large amount of water, and ridge mulching accelerates the growth of crops, which leads to increased water consumption. In the jointing stage, the change trend of soil water content in different soil layers showed significant differences with the heading stage and the filling stage. Mainly because the soil was irrigated during the jointing stage to meet the water consumption required for crop growth; meanwhile, the wheat root system developed mainly in the surface layer 0–10 cm. Therefore, the soil moisture content is consistent with the change trend of the seedling stage. In the crop heading period, the crop enters a period of large water demand. Ridging and mulching can promote the downward growth of wheat roots. Therefore, compared with traditional flat cropping, the soil moisture content is significantly reduced at 10–30 cm. These results show that during the jointing and heading stages of crops that require a lot of water, ridge mulching can effectively promote the growth of wheat and improve water use efficiency.

### Effect of different planting with film mulching on the phenological period of wheat

It can be seen from Table 4 that the winter wheat planted with different ridge film mulching methods can mature within the growth period of this variety. Among them, the T3 treatment with spraying liquid fertilizer has the shortest growth period of 232 days. Followed by the T2 treatment with black mulch, it has a growth period of 233 days. Traditional flat cropping treatment has the longest growth period of 235 days. Therefore, different ridge mulching methods can shorten the growth cycle of wheat, and the T3 treatment has the best effect.

**Table 4:** Different treatments of wheat in different growth periods (Unit: m/d/yy).

Stage	Sowing	Seedling	Jointing	Booting	Heading	Flowering	Mature	Growth period
CK	10/9/2020	10/18/2020	3/20/2021	4/7/2021	4/13/2021	4/23/2021	6/1/2021	235
T1	10/9/2020	10/18/2020	3/20/2021	4/6/2021	4/12/2021	4/22/2021	5/31/2021	234
T2	10/9/2020	10/17/2020	3/20/2021	4/6/2021	4/11/2021	4/21/2021	5/30/2021	233
T3	10/9/2020	10/17/2020	3/20/2021	4/5/2021	4/10/2021	4/20/2021	5/29/2021	232

### Effect of different mulching planting methods on photosynthetic efficiency of wheat

#### Effect of different mulching planting methods on wheat leaf area index

**Figure 6:**
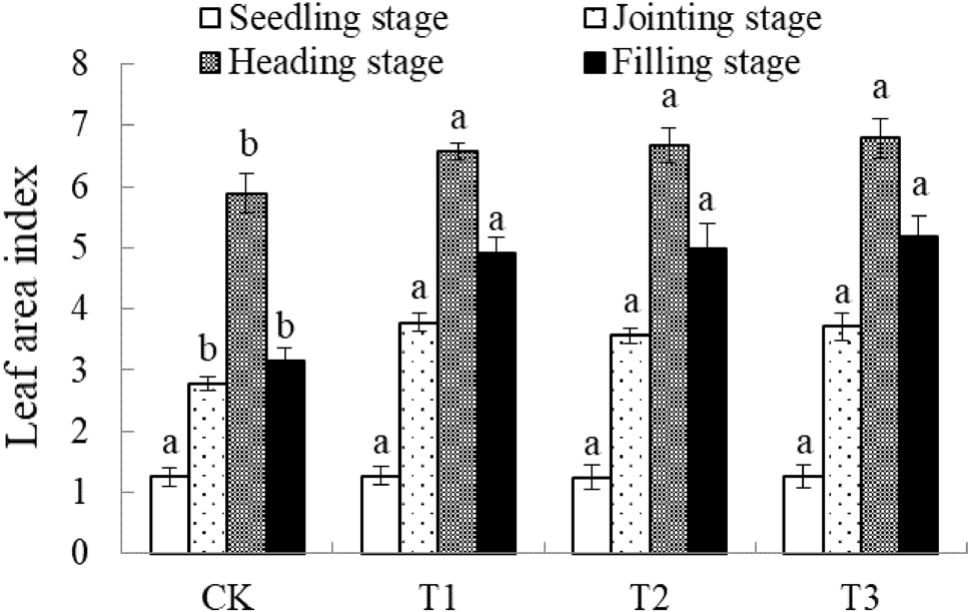
Changes of LAI of wheat in different stages of film mulching. Different lowercase letters in the figure represent significant differences between treatments (P < 0.05).

It can be seen from Fig. 6 that the change trend of the wheat LAI of different treatments is consistent. During the whole growth period, it showed a trend of increasing first and then decreasing, reaching the maximum at the heading stage, and gradually decreasing after heading until mature. There is no obvious difference in the leaf area index of each treatment at the seedling stage. The leaf area index of different ridge mulching treatments at the jointing stage is significantly higher than that of CK. Among them, the LAI of T1 treatment is the highest at 3.78, followed by T3 treatment with a leaf area index of 3.71, but there is no significant difference between them. At the heading stage, LAI increased significantly, and the LAI and CK of the ridge film mulching treatment reached a significant difference (*P* < 0.05). The specific performance is T3 > T2 > T1 > CK. The T3 treatment has the largest LAI, which is 6.79. Compared with CK, it increases by 15.28%. The LAI of each treatment in the growing period shows a slow downward trend; the LAI of the ridge film mulching treatment is higher than CK. The LAI of the T3 treatment is the largest which is 5.18. Followed by the T2 treatment, the LAI is 4.99. But the difference between the two is not significant; At the same time, compared with the heading date, CK decreased by 2.74%, while T1 ~ T3 respectively decreased by 1.66, 1.68 and 1.61%. In summary, ridge film mulching can effectively increase LAI so that it can maintain a higher LAI during the heading stage, and the decline of LAI in the later period will be slowed, which is conducive to the formation of high-efficiency LAI. Among them, the leaf area index of T3 treatment is the highest, and the decline is the most in the later period. slow.

#### Effect of different mulching planting methods on photosynthetic parameters of wheat flag leaves

**Figure 7:**
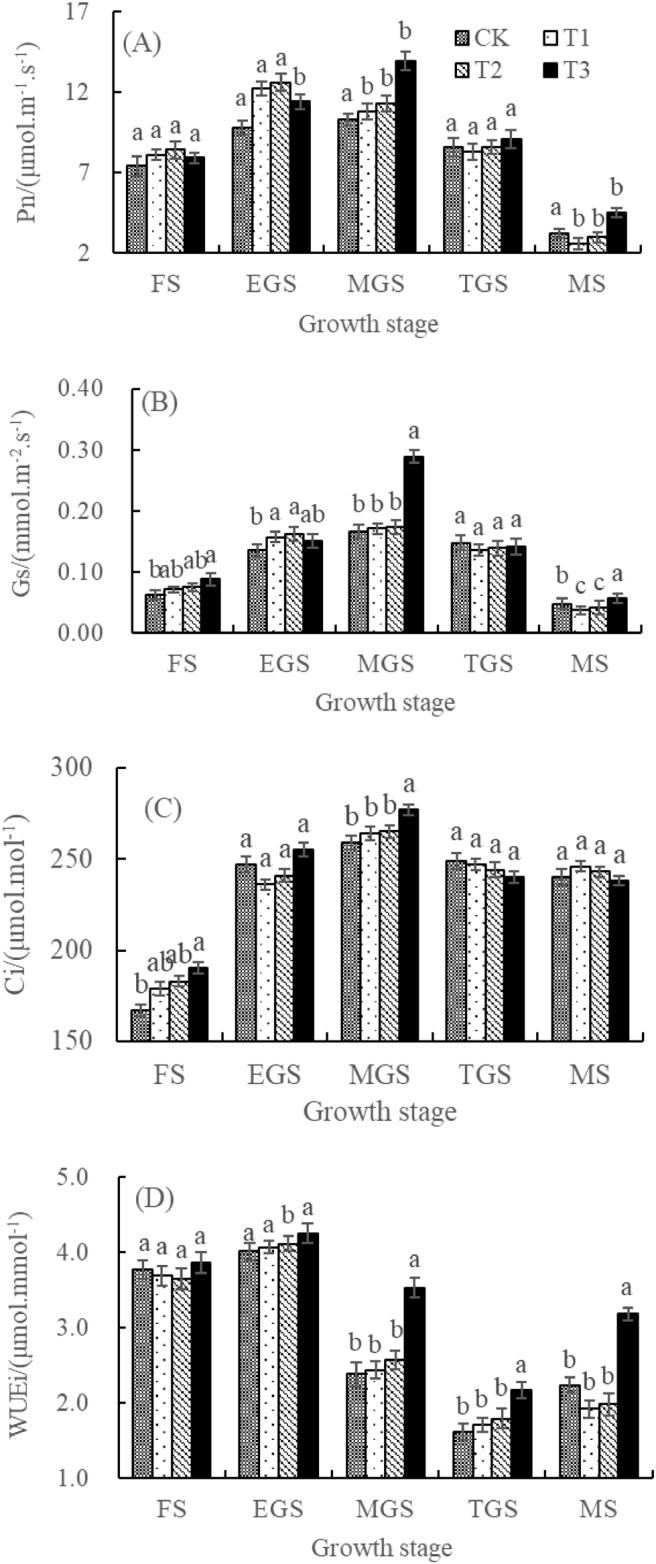
Effects of different covering models on photosynthesis of flag leaves during post-anthesis of winter wheat. *FS* Flowering stage, *EGS* Early grain-filling stage, *MGS* Middle grain-filling stage, *TGS* Terminal grain-filling stage, *MS* Milk stage. Different lowercase letters indicated significant difference among treatments at the same growth stage at P = 0.05 level. The same below.

Under different covering treatments, the Pn and Gs in flag leaves of wheat after anthesis show a trend of first increasing and then decreasing. T2 treatment reaches its peak in the early filling stage, while T3 treatment reached its peak in the middle filling stage (Fig. 7A,B). In the early stage of growth, the flag leaf Pn and Gs of T1 and T2 treatments are significantly higher than that of CK, but lower than that of CK in the middle and late stages of growth. T3 treatment is higher than that of CK throughout the entire growth period, and T1, T2, and T3 are 3.0% to 24.5%, 4.8% to 28.6%, and 5.8% to 41.9% higher than CK, respectively. This indicates that covering can improve flag leaf Pn and Gs, and the positive effect of T3 treatment runs through the entire post flowering growth period of wheat. T1 and T2 treatments play a positive effect in the early stage of growth, but in the middle and late stages of growth. In the later stage, there is a negative effect, T3 treatment has an advantage over T1 and T2 in improving the Pn and Gs of wheat flag leaves.

After anthesis, the flag leaf Ci of wheat under different treatments increases sharply and then slowly decreases, ultimately maintaining a high level (Fig. 7C). The changes in Ci from flowering stage to late filling stage can basically reflect the trend of wheat Pn changes (Fig. 7A,C). The changes in Ci from flowering to mid filling stage are significantly higher in T3 treatment than in T2, T1, and CK. They are 3.8% to 5.8%, 4.9% to 8.1%, and 3.2% to 13.8% higher, respectively. However, there are no significant differences among other treatments during the growth period. Compared with the other three cultivation modes, T3 significantly increased Ci early and middle grain-filling stage. It ensures the CO_2_ supply required for photosynthesis carboxylation under stomatal restriction conditions in wheat.

Under different coverage treatments, the flag leaf *WUE*i of wheat in semi-arid areas shows a trend of first increasing and then decreasing after flowering, with the lowest value in the late filling stage and gradually increasing afterwards (Fig. 7D). There is no significant difference in flag leaf *WUE*i between different treatments during flowering and early filling stages. However, the flag leaf *WUE*i of T3 treatments during mid filling, late filling, and milk ripening stages is significantly higher than that of T2, T1, and CK, which are 21.2% to 54.0%, 26.9% to 56.3%, and 34.0% to 48.3% higher, respectively. This indicates that T3 treatment significantly improved the flag leaf *WUE*i of dryland wheat in the middle and late stages of wheat breeding.

### Effects of different mulching planting methods on wheat biomass

It can be seen from Table 5 that the accumulation trend of above-ground biomass of wheat under different ridge film mulching treatments is consistent. Before the jointing stage, the biomass accumulation rate is relatively slow, but after the jointing stage, the biomass accumulation increases rapidly, and the accumulation rate gradually slows down after the heading and flowering, and finally the biomass accumulation reaches the maximum in the crop maturity stage. At the seedling stage, the aboveground biomass of T1 treatment was slightly lower than CK, while T2 and T3 treatments are slightly higher than CK, which increases by 1.2% and 2.7% respectively compared with CK, but the difference between treatments is not significant. The biomass accumulation of each ridge mulching treatment at the jointing stage is higher than that of CK, but the difference is not significant. By the heading stage, the biomass of each treatment accumulated rapidly, and the ridging and mulching treatments are significantly higher than that of CK; the biomass of different treatments is T3 > T1 > T2 > CK, and the biomass of T3 treatment is the largest, which is 7411.35 kg hm^−2^, it increases by 22.96% compared with CK, and it is significantly different from CK (P < 0.05). At the maturity stage, as the wheat began to enter the rapid stage of reproductive growth, the biomass accumulation rate slows down. However, the biomass accumulation of all ridge mulching treatments is higher than that of CK, and the evaluation is higher than that of CK. T1 ~ T3 treatments are respectively better than CK. The increase is 12.63%, 13.70% and 23.16%, among which the biomass of T3 treatment is the highest, reaching 8829.58 kg hm^−2^, which is significantly higher than other ridge mulching treatments and CK (P < 0.05). Comprehensive analysis shows that the ridging and mulching treatment can effectively increase the biomass of wheat. The main reason is that the ridging and mulching have the effect of retaining water and increasing moisture, which is beneficial to the growth and development of crops, and promotes the accumulation of biomass, which is beneficial to the growth of wheat. For the accumulation of nutrients, T3 has the best effect.

**Table 5:** Different treatments of wheat biomass in different periods (kg hm^−2^).

Treatment	Seedling stage	Jointing stage	Heading stage	Filling stage
CK	590.42a	2421.33a	6027.22c	7168.89c
T1	589.32a	2410.00a	7368.62ab	8074.58b
T2	597.56a	2564.89a	7159.25b	8151.21b
T3	606.33a	2551.74a	7411.35a	8829.58a

Different lowercase letters in the same column represent significant differences between treatments (P < 0.05).

### Relationship between biomass and LAI of wheat with different growth stages

**Figure 8:**
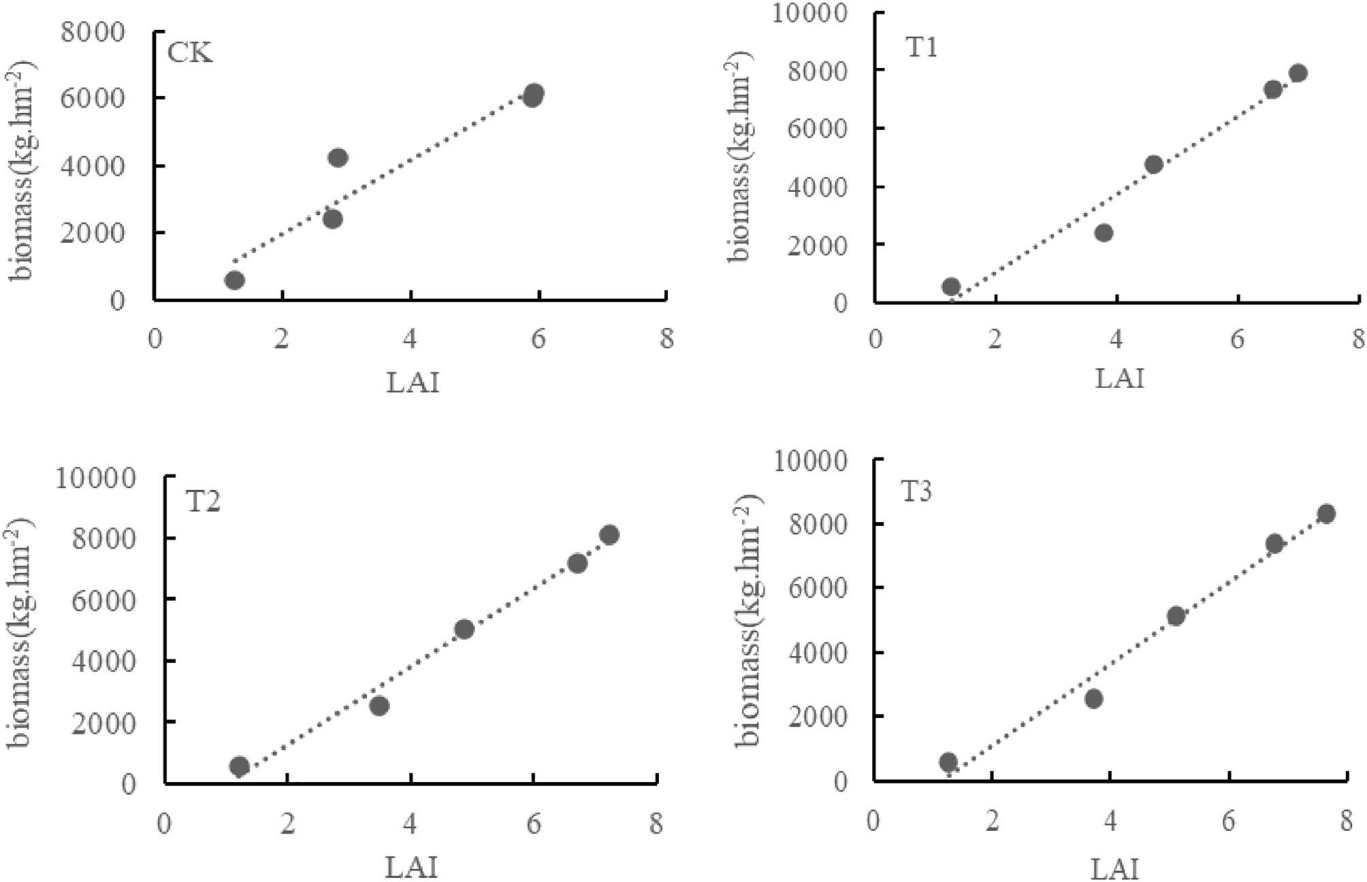
The correlation between LAI and biomass.

There is a linear positive correlation between LAI and biomass in wheat from seedling stage to flowering stage (Fig. 8). The correlation coefficient R values of different treatments were higher and significantly correlated (P < 0.05). The regression equation is shown in Table 6. All treatments showed strong positive correlations. The correlation between wheat biomass and LAI is close, with the highest R^2^ reaching 0.9857; CK has the worst tightness (R^2^ = 0.8994).

**Table 6:** Correlation between biomass and LAI in Wheat.

Treatment	Regression equation	R^2^	Correlation coefficient
CK	y = 1094.2x − 197.72	0.8994	0.9484
T1	y = 1335.8x − 1585	0.9633	0.9815
T2	y = 1268.8x − 1265.3	0.9857	0.9928
T3	y = 1263.7x − 1395.7	0.9816	0.9908

### Study the effects of different planting methods with film mulching on wheat yield and water use efficiency

It can be seen from Table 3 that different ridging and mulching treatments significantly affect the yield components and final yield of wheat. The number of ears of CK in the table is the highest. The main reason is that the ridging and mulching treatment has reduced the sowing area. Therefore, the number of ears formed by the traditional flat cropping treatment is higher than that of the ridge mulching treatment. The number of grains per ear and 1000-grain weight of the ridge film mulching treatment are significantly higher than CK, which effectively compensated for the loss caused by the reduction in area; Through the actual yield calculation of the final harvest, the ridge film mulching treatment has a significant increase in yield compared to CK. The analysis of wheat constituent factors shows that the number of ears and thousand-grain weight of different treatments are T3 > T2 > T1 > CK. The effect of T3 treatment is better than other treatments. The number of ears and thousand-grain weight are 24.44 and 41.00 g, respectively, compared with CK. It is increased by 19.3% and 5.4%; the difference in 1000-grain weight of each ridge and film mulching treatment is not significant, but the number of grains per ear and 1000-grain weight are significantly higher than CK (P < 0.05). Among them, the yield of T3 treatment reached 4098.25 kg hm^−2^. Followed by T2 treatment, it is 3837.78 kg hm^−2^, which respectively increases by 29.37% and 21.15% compared with CK. And they are significant differences with CK (P < 0.05), but there is a difference between the two Not obvious. Comprehensive analysis shows that the same treatment has the best effect on wheat yield components and final yield. T3 has the best effect. And the increase in yield of T3 is the result of increasing the number of grains per unit area and grain weight.

It can be seen from Table 7 that different film mulching significantly improved the water use efficiency of wheat, and the difference is significant from the CK. The water use efficiency of T3 treatment was the highest, increasing by 57.6% compared to CK, and there was a significant difference compared to other treatments (P < 0.05).

**Table 7:** Effect of different treatments on wheat yield components and yield.

Treatment	Spike number (× 10^4^‧hm^−2^)	Grain number	Thousand grain weight (g)	Yield (kg hm^−2^)	WUE [kg (mm^−1^ hm^−2^)]
CK	610.78a	20.48bc	38.91b	3167.78c	16.40d
T1	570.59b	19.76c	40.32a	35 49.20b	19.81c
T2	593.48ab	22.09b	40.41a	3837.78ab	22.52b
T3	605.88a	24.44a	41.00a	4098.25a	25.84a

Different lowercase letters in the same column represent significant differences between treatments (P < 0.05).

## Discusses

The adoption of surface covering cultivation measures can effectively reduce the evaporation of soil, thus improving the soil moisture^24,25^. In this study, the soil moisture content of traditional flat cropping during the seedling stage was the lowest; From the jointing stage to the filling stage, the soil moisture content of traditional flat cropping was higher than that of other ridge mulching treatments. Compared with CK, coverage mainly increases soil moisture in the early stages of growth, while soil moisture in some soil layers T3, T2, and T1 in the middle and late stages of growth is even slightly lower or no significant difference compared to CK. Other studies have also found similar results^26–29^. There are two possible explanations for these results. Firstly, in the middle and late stages, the transpiration water consumption (T) of treatments with larger canopies may be higher than that of CK^30,31^; Secondly, the conduction of water in the soil has resistance, and the equilibrium of water migration from high water potential areas to low water potential areas requires a certain length of time^32,33^. Studies have shown that the high yield of winter wheat covered cultivation is based on high water consumption^34^. At the same time, surface covering can effectively retain precipitation during the summer fallow period, replenish soil moisture, and maintain soil moisture balance^35^. This is consistent with the result that the soil moisture content in the film covering treatment is lower than that in the control treatment in this study.

A suitable soil environment is a key factor in ensuring the smooth progress of wheat leaf photosynthesis^36^. Covering can reduce soil surface evaporation, improve soil moisture, improve crop water utilization efficiency, and regulate the distribution of soil water at different growth stages of wheat^37^. The study on the photosynthetic potential of flag leaves of dryland winter wheat under different cultivation modes in this experiment showed that the pattern between treatments was T3 > T2 > T1 > CK. Among them, the effects of T1 and T2 treatments were not significant, while T3 had a significant effect in the middle and late stages of filling and from filling to milky maturity, which increased by 22.5% and 27.4% respectively compared to CK. This indicates that T3 can improve the photosynthetic potential, and the reason may be that T3 ensures relatively sufficient soil moisture conditions, Therefore, plants do not need to reduce leaf area for less transpiration, which may also be related to the transport and distribution of endogenous hormones involved in water^38^. At the same time, relatively sufficient soil moisture conditions ensure the supply of soil nitrogen to plants, thereby increasing the chlorophyll synthesis of wheat flag leaves in semi-arid areas^39^ and increasing the leaf area index of wheat. This is consistent with the results of this study. Ridge mulching effectively improves the LAI of wheat, maintaining a high LAI during the heading stage. Among them, the leaf area index of T3 treatment is the highest, and the decrease is the slowest in the later stage.

Covering cultivation increased the proportion of dry matter stored in wheat grains from post flowering vegetative organs. Therefore, covering cultivation can all increase grain weight^40^, which is consistent with the study of dryland winter wheat yield under different cultivation modes in this experiment. The yield table under different covering cultivation modes is T3 > T2 > T1 > CK, and T3 treatment increases yield by% compared to CK, with an increase in yield amplitude greater than T1 and T2. This may be due to the increased photosynthetic potential and leaf area index of T3 compared to uncovered open field cultivation.

During the jointing and heading stages when crops require a large amount of water, ridging and film covering can effectively promote wheat growth and improve water use efficiency. This is consistent with the study by Ye et al.^36^, which showed that the water use efficiency of wheat sown in furrows with ridging and plastic film mulching was significantly higher than that of traditional flat cropping wheat. The main reason is that plastic film covering can effectively retain soil moisture, increase soil water storage, reduce ineffective evaporation of soil moisture, coordinate the contradiction between water use and water demand for crop growth, and promote the utilization of deep soil moisture, thereby improving water use efficiency^37,38^.

Different ridge mulching methods can shorten the growth cycle of wheat and increase biomass and wheat yield. The T3 treatment with biodegradable film has the shortest growth period of 232 days. The main reason is that under the condition of plastic film mulching, it can increase soil temperature, promote crop growth, and shorten the growth period, especially the emergence period. For some crops, shortening the emergence period can produce early growth and increase yield^41^. Zhang et al.^42^ found that the yield of maize under mulching conditions increased significantly, and the yield increased significantly by 3800 kg hm^−2^. This is consistent with the results of this study. The yield of wheat was significantly increased by mulching treatment. The yield of T3 reached 4098.25 kg hm^−2^. It was an increase of 29.4%, compared with CK. Comprehensive analysis shows that different treatments significantly affect the growth cycle of wheat, yield components and the final yield, and the effect of covering with liquid film is the best. Other studies shown that in the Loess Plateau region, the increase in production of environmentally friendly plastic films was lower than that of traditional plastic films, and the economic benefits were lower^43^. This is inconsistent with the results of this study. This may be because there is a serious water shortage in the Loess Plateau region, and the water retention performance of different covering materials has a significant impact on yield; However, this study is in a semi-arid region, and the results indicate that there is no significant difference in the impact of different covering materials on soil moisture content. However, environmentally friendly plastic film can improve indicators such as leaf area index, resulting in a higher final yield. Due to the limited literature, further research is needed for specific reasons.

## Conclusions

1. Mulching treatment effectively increased the soil moisture content of different soil layers during the seedling stage, but the soil moisture content of each treatment was lower than CK from the jointing stage to the mature stage.
2. Ridge forming and film covering treatment can effectively affect the photosynthetic parameters of wheat flag leaves, increase the leaf area index of wheat, and ultimately improve the photosynthetic efficiency of wheat. Among them, T3 treatment has the best effect.
3. Various covering treatments in the Loess Plateau area can effectively improve soil water use efficiency, increase the number of grains per spike and thousand grain weight of wheat, and achieve significant yield increase. The yield increasing effect of each treatment is in the order of T3 > T2 > T1 > CK.

Therefore, this study recommends selecting the liquid plastic film covering mode (T3 treatment) in the semi-arid loess plateau area. This model not only effectively improves crop yield, but also reduces the pressure on soil ecosystems, thereby effectively alleviating the increasingly serious problem of white pollution.

## Data Availability

All data generated or analyzed during this study are included in this published article. Wheat (Triticumaes-tivum) Xiaoyan 22 was purchased from Shaanxi Fuping County Seed Industry Co., Ltd. The material source complies with relevant institutional, national, and international guidelines and legislation. The experimental land is provided by Shaanxi Land Engineering Construction Group. We have obtained the permission from the landowner.

## References

[1] Yang YH, Ding JL, Zhang YH, Wu JC, Zhang JM, Pan XY, Gao CM, Yue W, He F (2018). Effects of tillage and mulching measures on soil moisture and temperature, photosynthetic characteristics, and yield of winter wheat. Agric. Water Manage.

[2] Tan YZ, He J, Yue WZ, Zhang L, Wang QR (2017). Spatial pattern change of the cultivated land before and after the second national land survey in China. J. Nat. Resour..

[3] Hao HB, Liu JF, Chen XW, Wang ZH (2019). Straw mulch as an alternative to plastic film mulch: Positive evidence from dryland wheat production on the Loess Plateau. Sci. Total Environ..

[4] He G, Wang Z, Li FC, Dai J, Li Q, Xue C, Cao HB, Wang S, Malhi SS (2016). Soil water storage and winter wheat productivity affected by soil surface management and precipitation in dryland of the Loess Plateau, China. Agric. Water Manage.

[5] Yang LL, Zhang WP (2019). Impact of climate change on agricultural meteorological disasters in fuping county and defensive measures. Modern Agric. Sci. Technol..

[6] Deng XP, Shan L, Zhang H, Turner NC (2006). Improving agricultural water use efficiency in arid and semiarid areas of China. Agric. Water Manage.

[7] Zhang P, Wei T, Wang HX, Wang M, Meng XP, Mou SW, Zhang R, Jia ZK, Han QF (2015). Effects of straw mulch on soil water and winter wheat production in dry land farming. Sci. Rep..

[8] Gao H, Yan C, Liu Q, Ding W, Chen B, Li Z (2019). Effects of plastic mulching and plastic residue on agricultural production: a meta-analysis. Sci. Total Environ..

[9] Li FM, Li XG, Javaid MM, Li F, Li XG, Javaid MM, Ashraf M, Zhang F (2020). Ridge-furrow plastic film mulching farming for sustainable dryland agriculture on the Chinese loess plateau. Agron. J..

[10] Yang YF, Wu FQ, Xu N, Duan HT (2020). Effects of furrow-ridge mulching with different furrow-ridge ratios on yield and water use efficiency of Wolfberry. J. Soil Water Conserv..

[11] Qin YQ, Chai YW, Li R, Li YW, Ma JT, Cheng HB, Chang L, Chai HX (2022). Evaluation of straw and plastic film mulching on wheat production: A meta-analysis in Loess Plateau of China. Field Crops Res..

[12] Chen XW, Zhao HB, Liu J, Mao AR (2020). Winter wheat nitrogen utilization under different mulching practices on the Loess Plateau. Agron J..

[13] Qin YQ, Cheng HB, Chai YW, Ma JT, Li R, Li YW, Chang L, Chai SX (2022). Increasing effects of wheat yield under mulching cultivation in northern of China: A meta-analysis. Sci. Agric. Sin..

[14] Hu Q, Li X, Gonçalves JM, Shi HB, Tian T, Chen N (2020). Effects of residual plastic-film mulch on field corn growth and productivity. Sci. Total Environ..

[15] Sun DB, Li HG, Wang EL (2020). An overview of the use of plastic film mulching in China to increase crop yield and water. Natl. Sci. Rev..

[16] Qi R, Jones DL, Li Z, Liu Q, Yan CR (2020). Behavior of microplastics and plastic film residues in the soil environment: A critical review. Sci. Total Environ..

[17] Wan Y, Wu C, Xue Q, Hui XMN (2019). Effects of plastic contamination on water evaporation and desiccation cracking in soil. Sci. Total Environ..

[18] Wang L, He XW, Hu C, Wang XF, Liu Q, Yan CR, Ding JP (2021). Effects of degradation properties of biodegradable plastic mulches on soil temperature, humidity and cotton yield in Southern Xinjiang. Agric. Res. Arid Areas.

[19] Min WH, Wang CL, Wang LW, Yi TH, Bian JJ, Zhi M, Sun QH, Su JJ, Zhao XL (2022). Effects of biodegradable film raw material particles on soil properties, wheat growth, and nutrient absorption and transportation. Environ. Sci..

[20] Jin J, Lu Y (2021). Research on the factors influencing the demand for agricultural biodegradable plastic films. Ecol. Econ..

[21] Wang ZX, Guo BQ, Li JZ (2015). Preliminary study on the application of liquid film in flax production. Inner Mongolia Agric. Sci. Technol..

[22] Zhang CY, Yang XM (2008). Affects of liquid film on growth and yield of maize. J. Qingdao Agric. Univ. Nat. Sci..

[23] Guo ZH, Zhang XD, Huang LL, Ju GS, Chen JQ (2006). Solar energy and water utilization of Quercus mongolica, a deciduous broad leaf tree, in different light regimes across the edge of a deciduous broad-leaved forest. Acta Ecol. Sin..

[24] Li R, Hou XQ, Jia ZK, Han QF, Ren XL, Yang BP (2013). Effects on soil temperature, moisture, and maize yield of cultivation with ridge and furrow mulching in the rainfed area of the Loess Plateau, China. Agric. Water Manag..

[25] Zhang SL, Sadras V, Chen XP, Zhang FS (2013). Water use efficiency of dryland wheat in the Loess Plateau in response to soil and crop management. Field Crops Res..

[26] Ram H, Dadhwal V, Vashist KK, Kaur H (2013). Grain yield and water use efficiency of wheat (*Triticum aestivum* L.) in relation to irrigation levels and rice straw mulching in North West India. Agric. Water Manage.

[27] Yan QY, Dong F, Yang F, Lu JX, Li F, Zhang JC, Dong JL, Li JH (2019). Improved yield and water storage of the wheat-maize rotation system due to double-blank row mulching during the wheat stage. Agric. Water Manage.

[28] Liu G, Zuo Y, Zhang Q, Yang L, Zhao E, Liang L, Tong Y (2018). Ridge-furrow with plastic film and straw mulch increases water availability and wheat production on the Loess Plateau. Sci. Rep..

[29] Zhang P, Wei T, Han QF, Ren XL, Jia ZK (2020). Effects of different film mulching methods on soil water productivity and maize yield in a semiarid area of China. Agric. Water Manage.

[30] Lin W, Liu WZ, Zhou SS, Liu CF (2019). Influence of plastic film mulch on maize water use efficiency in the Loess Plateau of China. Agric. Water Manage.

[31] Wang YP, Li X, Zhu J, Fan CY, Kong XJ, Turner NC, Siddique KHM, Li FM (2016). Multisite assessment of the effects of plastic-film mulch on dryland maize productivity in semiarid areas in China. Agric. Meteorol..

[32] Bittellia M, Venturaa F, Campbellb GS, Snyder RL, Gallegati F, Pisa PR (2008). Coupling of heat, water vapor, and liquid water fluxes to compute evaporation in bare soils. J. Hydrol..

[33] Joel A, Messing I (2001). Infiltration rate and hydraulic conductivity measured with rain simulator and disc permeameter on sloping arid land. Arid Land Res. Manage.

[34] Chen YZ, Chai SX, Tian HH, Chai YW, Li YW, Chang L, Cheng HB (2019). Straw strip mulch on furrows improves water use efficiency and yield of potato in a rain fed semiarid area. Agric. Water Manage.

[35] Chai SX, Yang CG, Zhang SF, Chen HH, Chang L (2015). Effects of plastic mulching modes on soil moisture and grain yield in dryland winter wheat. Acta Crop Sci..

[36] Ye YL, Feng YT, Xu JX, Zhang RZ, Hu CL, Lei T, Zhang SL (2020). Effect of plastic film mulching on wheat yield and water use efficiency in South of Loess Plateau. Northw. Agric. J..

[37] Qian R, Liu Y, Guo R, Yang L, Liang X, Zhang P, Ren XL, Jia ZK (2021). Effects of straw returning to field and plastic film mulching on soil water, temperature and maize yield in upland fields. Northw. Agric. J..

[38] Qian CX, Chen JK, Yu LY, Chen JL, Xu CX (2021). Effects of different film mulching cultivation on water use efficiency and yield of early spring potato in low latitude plateau. Agric. Eng. Technol..

[39] Yang R, Tian CY, Mai WX (2016). Characteristics of root development in cotton suffering presenility under drip irrigation and film mulch in Xinjiang Autonomous Region. J. Plant Nutr. Fertil..

[40] Chen YH, Cheng HB, Liu Y (2019). Effect of covering on photosynthetic characteristics of flag leaf during post-anthesis and yield in winter wheat. Agric. Res. Arid Areas.

[41] Li BY, Chen JP, Liu AN, Wu PJ, Wang HZ (2021). Soil warming changed growth, yield and water consumption of winter wheat. J. Irrigat. Drainage.

[42] Zhang HF, Wang W, Wei SZ (2015). Influence of plastic mulching on soil properties and maize yield in Linzhi, Tibet. J. Northw. Sci. Technol. Univ. Agric. For. Nat. Sci. Ed..

[43] Qin YQ (2022). Effect of Mulching Cultivation Technology on Wheat Yield and ecology in the Loess Plateau: A Meta-analysis.

